# Utility of a CAD model for estimation of vascular involvement and R0 resection in pancreatic ductal adenocarcinoma

**DOI:** 10.1097/JS9.0000000000003048

**Published:** 2025-07-17

**Authors:** Tianyu Zhao, Yu Wen, Yajie Chen, Zihang Huang, Xixi Jiang, Keke Si, Heshui Wu, Xin Yang, Xin Li

**Affiliations:** a School of Electronic Information and Communications, Huazhong University of Science and Technology, Wuhan, China; b Department of Radiology, Union Hospital, Tongji Medical College, Huazhong University of Science and Technology, Wuhan, China; c Hubei Provincial Clinical Research Center for Precision Radiology & Interventional Medicine, Wuhan, China; d Hubei Key Laboratory of Molecular Imaging, Wuhan, China; e School of Engineering, The Hong Kong University of Science and Technology, Hong Kong, HK; f Department of Radiology, The Affiliated Cancer Hospital of Zhengzhou University & Henan Cancer Hospital, Zhengzhou, Henan, China; g Department of Pancreatic Surgery, Union Hospital, Tongji Medical College, Huazhong University of Science and Technology, Wuhan, Hubei Province, China

**Keywords:** Computer-Aided Diagnosis, Image Features Extraction, Image Segmentation, Pancreatic Ductal Adenocarcinoma, Vascular Involvement

## Abstract

**Background:**

The current NCCN guidelines for evaluating vascular involvement in pancreatic ductal adenocarcinoma (PDAC) includes limited evaluation metrics, coarse granularity, and significant inter-observer variability.

**Purpose:**

This study aimed to develop a computer-aided diagnosis (CAD) model to predict PDAC vascular involvement and R0 Resection by image features extraction.

**Materials and Methods:**

This study retrospectively included consecutive patients with PDAC after preoperative CT between 2020 and 2022. Based on DoDNet, a segmentation network of PDAC, pancreas and vessels was trained and performed on CT images to extract image features, which characterized morphology, special relationship and texture information. The intraoperative and postoperative histopathological findings were used as reference standards. The assessment of vascular involvement by experienced pancreatic radiologist was the comparison. Features were selected by Fisher Score ranking and Wald Chi-Square Test and merged to predict PDAC vascular involvement.

**Results:**

A total of 87 patients (mean age: 62.53 ± 7.73 years; 55 males) were evaluated. All patients had histopathologically confirmed PDAC with intraoperative vascular involvement scores, 61 of which had postoperative margin pathology information. A total of 229 image features were extracted. The AUC of CAD model with 3 features (tumor-vessel encasement angle $\theta_{encase}$, vascular deformation risk score $RS_{deform}$, tumor morphology risk score $RS_{tumor}$) reached (CA) and partially exceeded (CHA, SMA, PV, SMV) the radiologists’ predictions of vascular involvement, where $\theta_{encase}$ and $RS_{deform}$ were significant risk factors respectively for involvement of the 5 major vessels (SMA, CA, CHA, PV, SMV, P < .01) and veins (SMV, PV, P < .05). Specifically, CAD model showed sensitivity comparable to or higher than radiologists, but lower specificity. The CAD reached radiologists’ predictions of R0 resection.

**Conclusions:**

The proposed CAD model extracts CT image features, serving as an objective, reproducible, and accurate tool for predicting PDAC vascular involvement and R0 resection.

## Introduction

Pancreatic ductal adenocarcinoma (PDAC) is a highly malignant and fatal tumor of the digestive system with a poor prognosis, having an overall 5-year survival rate of about 10%^[1,2]^. Surgical resection is the most effective treatment for pancreatic cancer, but only 15-20% of patients are eligible for surgery at the time of diagnosis^[3]^. In the absence of distant metastases, the tumor-vessel relationship on CT images of PDAC patients is a crucial factor guiding clinical treatment decisions and is essential for selecting the appropriate treatment method for patients. Enhanced CT is the preferred imaging modality for assessing the relationship between PDAC and adjacent vessels and analyzing resectability^[4]^, focusing primarily on the circumferential contact degree between the tumor and surrounding vessels and changes in vascular morphology^[5–8]^. For example, the National Comprehensive Cancer Network (NCCN) criteria classifies pancreatic tumors as showing abutment or encasement of vessels on the basis of a threshold of 180°of tumor contact with the vessel circumference^[5]^. However, this standard in clinical applications is affected by subjective judgment and experience differences among observers, scanning techniques, and other patient-related factors. Studies have shown that even experienced radiologists exhibit significant inter-observer variability in evaluating the relationship between PDAC and adjacent vessels and in assessing resectability^[9,10]^.

The current NCCN evaluation system includes few evaluation metrics, has coarse granularity and poor quantifiability, making it crucial to refine and enrich the evaluation metrics. Research indicates that incorporating additional evaluation metrics, such as vascular involvement length and tumor-vessel contact area, helps more accurately assess vascular involvement^[6,11]^. However, newly proposed imaging features often face issues with generalizability, and an excessive number of observed metrics can lead to difficulties in clinical implementation and widespread adoption.

Compared with visual image analysis alone, the use of radiomics can capture additional information about the tumor and its surrounding environment, providing more observational metrics for the clinic. Computer-aided diagnosis (CAD) leverages automatic image processing (registration and segmentation) to exploit image features^[12–14]^, including texture features^[15–21]^ and morphological features^[19,22,23]^, which provide more observation indicators to supplement the evaluation system and reduce the impact of inter-observer variability. However, current CAD models are often constructed on specific datasets, which limit their adaptability in imaging equipment and qualities^[16]^.

However, the current studies using radiomics or artificial intelligence methods in predicting vascular involvement and surgical margin of PDAC are relatively limited, and most of them are limited to the evaluation of single vessel involvement (Superior Mesenteric-Portal Vein, Superior Mesenteric Artery), and mainly focus on the feature of tumor-vessel ring contact^[11,21,22,24,25]^. Some studies take the results of preoperative evaluation results of doctors as he reference standard of vascular involvement^[11]^, which is somewhat subjective. Radiomics requires manual segmentation of the tumor and the surrounding blood vessels to extract information, which requires a large amount of human resources and is not suitable for clinical application. In addition, these models rely on hundreds of hand-made radiomics features, making the final decision process of the models difficult to interpret and reproduce.

To overcome these limitations, this study aims to construct a CAD model for more objective, reproducible, automated and interpretable quantitative assessment method to predict vascular involvement and R0 resection in patients with PDAC. We declare compliance with the TITAN Guidelines 2025 governing the declaration and use of artificial intelligence^[26]^.

## Material and method

### Study patient

**Figure 1.**
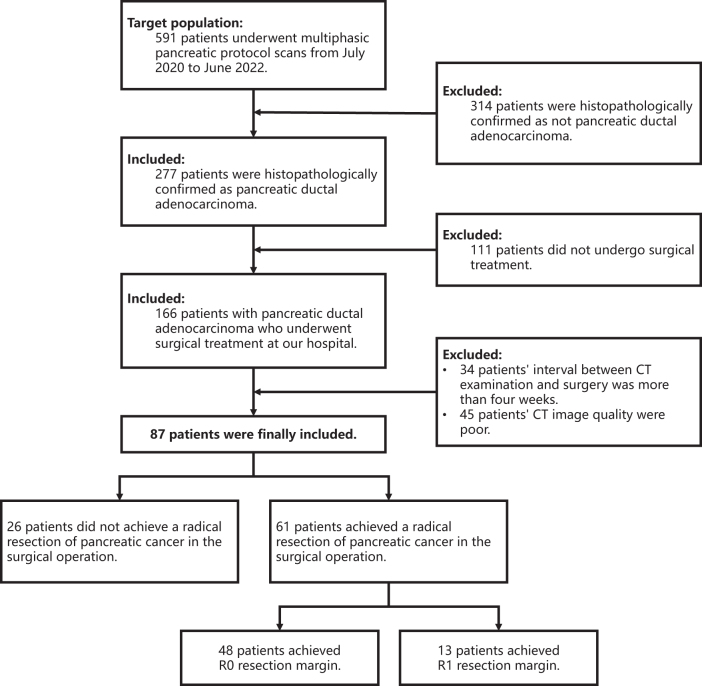
Patient accrual flowchart based on recommended standards for reporting diagnostic performance. PDAC = pancreatic ductal adenocarcinoma.

We retrospectively collected all patients with PDAC confirmed by histopathology who underwent multi-phase contrast-enhanced dual-energy CT (DECT) scanning using the pancreatic protocol (detailed below) from July 2020 to June 2022. Participants from a previous study^[27]^ were included in this study. The patient selecting process is shown in (Fig. 1), with the exclusion criteria as follows: (a) patients who did not undergo surgery; (b) patients with a time interval of more than 4 weeks between surgery and CT examination; (c) patients with poor image quality (Excessive image artifacts preclude diagnostic use; or even if diagnosable, the five key vessels for evaluation have blurred/indistinct margins). (d) multiple PDAC lesions. Clinical information for each patient was collected, including demographic characteristics (age, gender, BMI), neoadjuvant therapy information, tumor location information, tumor markers (CA-199, CA-125, CEA), surgical methods, intraoperative peripancreatic vascular involvement information, and postoperative pathological margin and tumor differentiation information.

**HIGHLIGHTS**

- Trained an image segmentation model on public datasets to segment multiple abdominal organs.
- Designed several imaging features for vascular invasion.
- The proposed cad model reached or exceeded radiologists’ diagnostic ability for pdac vascular involvement.

### CT protocol

All patients underwent multi-phase DECT scanning according to the pancreatic protocol on Philips IQon Spectral CT. This included a pancreatic parenchymal phase and a portal venous phase. Scanning parameters are summarized in Supplemental Digital Content Appendix A, available at: http://links.lww.com/JS9/E676.

### Image analysis and reference standard

The analysis and evaluation were conducted jointly by two experienced pancreatic radiologists (with 6 and 20 years of abdominal radiology diagnostic experience, respectively) without knowledge of the surgical and pathological results. In cases of disagreement, a third, more experienced pancreatic radiologist (with 30 years of abdominal radiology diagnostic experience) provided the final assessment. Each patient was evaluated for the relationship between the tumor and the five major peripancreatic vessels (celiac artery, CA; common hepatic artery, CHA; superior mesenteric artery, SMA; portal vein, PV; superior mesenteric vein, SMV). According to NCCN standards, the tumor-vessel relationship was classified as: no contact, abutment (contact ≤180°), or encasement (contact >180° or vascular deformation)^[5]^. The radiologists also recorded the presence of occlusion or tumor thrombus formation in PV or SMV.

Surgeons assessed vascular involvement using a combination of visualization and palpation, and documented findings in the surgical records as a reference standard for vascular involvement. According to the surgical records, the surgical presentation of the tumor-vessel relationship was categorized as follows: 1) no contact or adhesion; 2) adhesion but separable; 3) difficult to separate with suspected infiltration; 4) clearly invaded. The first two categories were defined as no intraoperative vascular invasion, while the latter two were defined as intraoperative vascular invasion. For patients undergoing PDAC resection, a retrospective analysis of pathological reports classified the pathological margin status as R0 (all resection margins microscopically negative) or R1 (tumor cells within 1 mm of the resection margin)^[28]^. Most surgeons will specially mark and stain the suspicious vascular invasion sites during surgery, and ask pathologists to focus on sampling to ensure the spatial consistency between the intraoperative suspected area and the pathological evaluation area.

Based on tumor-vascular relationships, we use “encasement” and “abutment/encasement” as radiologists’ predictor for vascular involvement to compare them with CAD model’s prediction.

### Partial-label segmentation algorithms based on DoDNet

**Figure 2.**
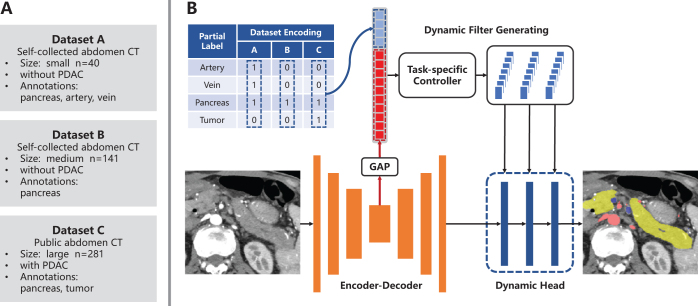
Datasets used in training stage and the structure of adopted segmentation network. (A) 3 collected partial labeled datasets of enhanced CT scans; (B) Segmentation network (DoDNet), composed of an encoder, a task encoding module, a dynamic filter generation module, and a dynamic segmentation head. The kernels in the dynamic head are conditioned on the input image and its labels.

**Figure 3.**
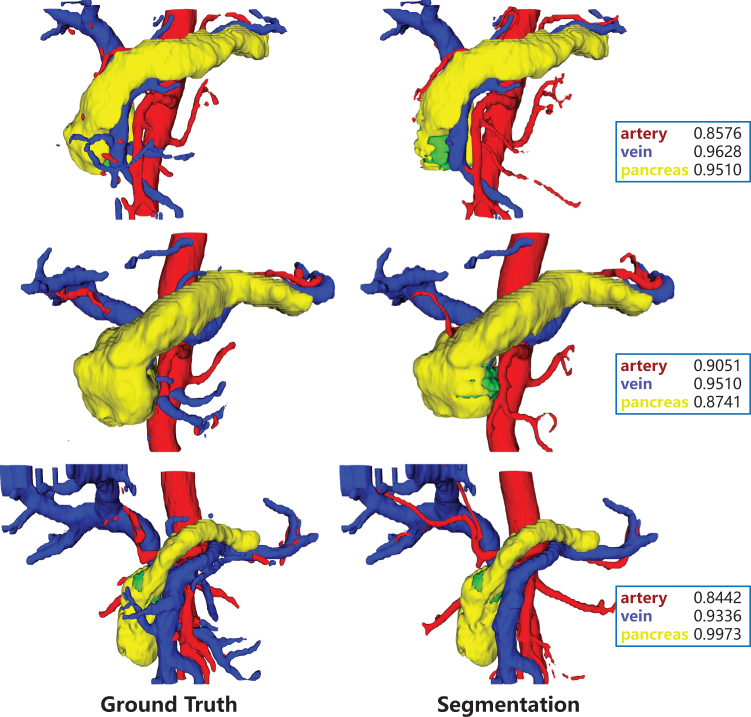
The segmentation masks of our proposed network (Segmentation, right column) versus the segmentation masks manually adjusted by doctors (Ground Truth, right column), with the dice coefficients for the segmentation targets of the showcased case marked in boxes.

**Figure 4.**
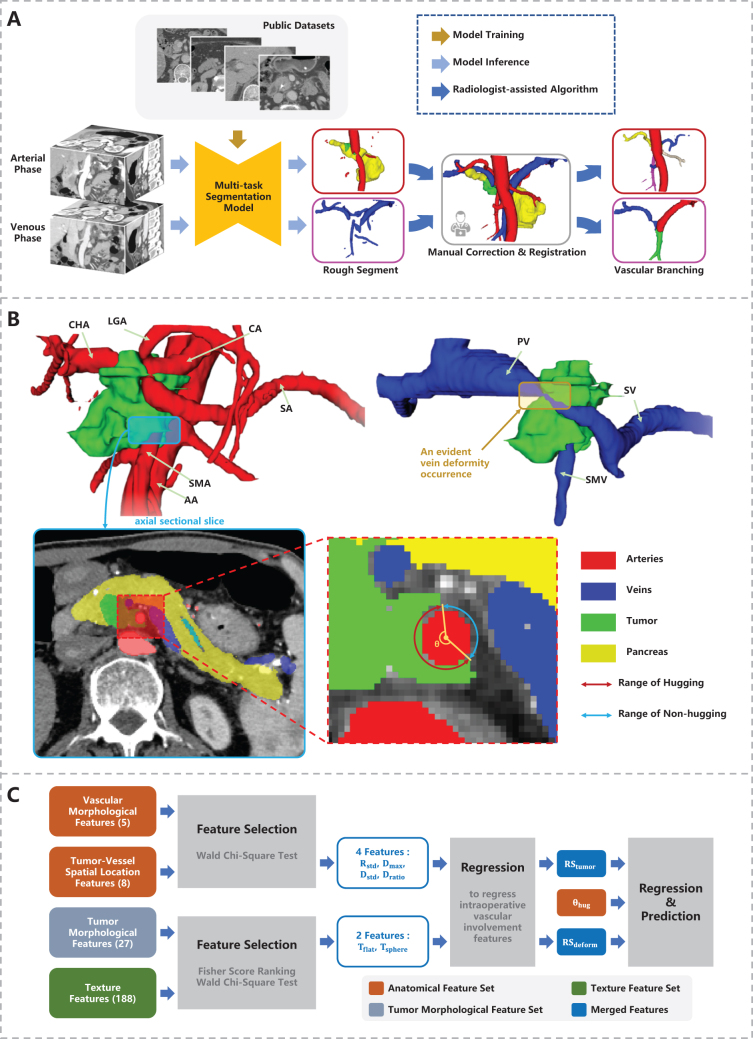
Schematic overview of the segmentation network and feature extraction process. (A) A multi-task segmentation network is trained on 4 public datasets and utilized for inference on the dataset of our work. The segmentation mask is generated from CT images of 2 phases, then corrected, registered and spilt into branches manually. (B) An example analysis of contrast-enhanced CT image from a 62-year-old man with pancreatic ductal adenocarcinoma in the head of the pancreas and without neoadjuvant therapy. The intraoperative pathology report showed CA, CHA, SMA, PV and SMV were all involved. And therefore, the tumor was considered unresectable by a consensus of two radiologists. The axial sectional slice form artery (left) shows the encasement angle $\theta_{encase}>180^{\circ }$. An evident occurrence of vein deformity (right, a typical feature of vein involvement) is pointed out in the 3D view of veins (right). (C) Feature selection and merging pipeline.

As shown in (Fig. 4(A)), to utilize the partial-annotated, multi-phase, multi-center public datasets for CT images segmentation of this study, we employed a network based on DoDNet^[29]^. Compared to other networks (like U-Net and ResNet), by encoding annotations information of datasets into the network, DoDNet better adapts to inconsistencies in annotations and significant domain shifts in the collected datasets, offering superior performance on unseen data and greater robustness across imaging environments. The network was trained on three different abdominal CT datasets simutaneously, each of which contains pixel-level annotations for only a portion of the segmentation targets. The network structure is illustrated in (Fig. 2), and see dataset details in Supplemental Digital Content Appendix B, available at: http://links.lww.com/JS9/E676. The network was implemented using PyTorch 3.6, and was trained for 400 epochs using a NVIDIA RTX 3090 GPU. The average dice coefficients on test datasets are as follows: pancreas (0.872 dataset A,B,C), PDAC (0.557, dataset C), veins (0.753, dataset A). We generated segmentation masks on our target dataset by segmenting pancreas, PDAC and veins in the portal venous phase, and arteries in the pancreatic parenchymal phase. These segmentation masks were then refined by an experienced pancreatic radiologist to generate the ground truth (GT), as shown in (Fig. 3).

Segmentation masks on 2 phases were registered and fused with deedsBCV^[30,31]^. To extract features from vascular branches independently, we designed a vascular branching method based on vascular skeletonization (see Supplemental Digital Content Appendix D, available at: http://links.lww.com/JS9/E676 for details), which subdivides segmentation masks based on anatomical structures of the vessels.

### Image feature extraction

We designed an anatomical feature set of 14 to characterize morphology and tumor-vessel spatial relationships (examples shown in Fig. 4(B)). Additionally, we performed high-throughput extraction of features using pyradiomics^[32]^ to construct a tumor morphology feature set of 27 and a texture feature set of 188 respectively (see Supplemental Digital Content Appendix C, available at: http://links.lww.com/JS9/E676). In summary, we extracted a total feature set of 229 each case, with some important features listed in Table 2.

### Statistical analysis

All statistical analyses were conducted using R software (version 4.1.2) and IBM SPSS Statistics software (version 27). The p<.05 was considered statistically significant. In the reported results, vascular involvement and R1 margin status were reported as positive. The performance of the CAD model was summarized using sensitivity, specificity, positive predictive value, negative predictive value, and AUC. The Wilcoxon rank-sum test assessed the distribution of features. Cohen’s kappa analysis evaluated inter-observer consistency. The McNemar test compared the differences in sensitivity and specificity between predictions of CAD model and radiologists, while the DeLong test compared the differences in AUC values between the two.

## Results

### Characteristics of study patients

As shown in the selecting flowchart (Fig. 1), 87 patients (mean age: 62.53 ± 7.73 years; 55 males) were included in this study, with 61 patients having postoperative pathological margin results. Patient demographic information is shown in Table 1.

**Table 1:** Characteristics of Study Patients

Characteristic	Value
Intraoperative (n = 87)	
Gender	
Male	55 (63.2%)
Female	32 (36.8%)
Age (years)	62.53 ± 7.73
BMI	21.83 (20.31, 23.92)
Location of primary tumor	
Head/Neck	54 (62.1%)
Body/Tail	33 (37.9%)
Neoadjuvant therapy	9 (10.3%)
Non neoadjuvant therapy	78 (89.7%)
Tumor Markers	
CA-199 (U/mL)	267.40 (37.80, 1200.00)
CA-125 (U/mL)	16.05 (10.55, 30.80)
CEA (μg/L)	3.25 (2.02, 5.65)
Interval Time Between Surgery and CT Scan (days)	7.0 (3.5, 10.0)
Vessel involvement at surgical	
Celiac Artery (CA)	8 (9.2%)
Common Hepatic Artery (CHA)	12 (13.8%)
Superior Mesenteric Artery (SMA)	8 (9.2%)
Portal Vein (PV)	22 (25.3%)
Superior Mesenteric Vein (SMV)	31 (35.6%)
Surgical method	
Pancreaticoduodenectomy	40 (46.0%)
Distal pancreatectomy	21 (24.1%)
Palliative surgery/Exploratory laparotomy	26 (29.9%)
Postoperative (n = 61)	
Resection Margin Status	
R0	48 (78.7%)
R1	13 (21.3%)
Differentiation Grade	
Well Differentiated	4 (6.6%)
Moderately Differentiated	41 (67.2%)
Moderately-Poorly Differentiated	10 (16.4%)
Poorly Differentiated	6 (9.8%)

Note—Data in parentheses are percentages or interquartile ranges. Data in brackets are the test used to perform statistical analysis. IQR = interquartile range, CA = Celiac Artery, CHA = Common Hepatic Artery, SMA = Superior Mesenteric Artery, PV = Portal Vein, SMV = Superior Mesenteric Vein.

The total number of data in the Resection part is n = 61, on which all percentages are based. The values presented in this part represent the number of patients with intraoperative vascular involvement and postoperative margins classified as R0 or R1. The counterpart values for cases with vessels uninvolved can be inferred from the total number (n = 61).

**Table 2:** Radiomics of Relationship of Perivascular Tissue and Primary Tumor

Feature Name	Definition	Mathematical Definition	Reasoning
**Vascular Morphological Features**			
Minimum Vessel Radius* $R_{min}$	Minimum, maximum, mean and standard deviation of the sequence of radius at every voxel from a single skeleton edge	$R_{min}=min\left(R^{n}\right)$ where $R^{n}$ is a list containing vessel radius at every voxel from a skeleton edge with a length of n voxels	
Maximum Vessel Radius* $R_{max}$	...	$R_{max}=max\left(R^{n}\right)$	
Mean Vessel Radius* $R_{mean}$	...	$R_{mean}=\frac{\sum \left(R^{n}\right)}{n}$	
Vessel Radius Range* $R_{range}$	...	$R_{range}=R_{max}-R_{min}$	
Vessel Radius Standard Deviation* $R_{std}$	...	$R_{std}=\sqrt{\frac{\sum \left({\left(R-R_{mean}\right)}^{2}\right)}{n}}$	
**Tumor Morphological Features**			
Tumor Surface Area $T_{surface}$	...		
Tumor Volume $T_{volume}$	...		
Tumor Sphericity $T_{sphere}$	The spherical shape of the tumor, with values ranging from 0 to 1, where 1 indicates a perfect sphere.	$T_{sphere}=\frac{\pi^{1/3}\cdot {\left(6\cdot T_{volume}\right)}^{2/3}}{T_{surface}}$	
Tumor Compactness $T_{compact}$	The compactness of the tumor region, with higher values indicating a more compact shape.	$T_{compact}=T_{surface}/T_{volume}$	
Tumor Maximum 3D Diameter $T_{maxD}$	The maximum diameter of the tumor, defined as the straight-line distance between the two furthest voxels on the tumor surface.	$T_{maxD}=\underset{v_{i},v_{j}\in S}{max}||v_{i}-v_{j}||$ where S is the surface area of the tumor, $v_{i}$ and $v_{j}$ are voxels on the surface	
Tumor Major Axis Length $T_{majorAxis}$	The longest linear dimension of the tumor along its primary axis.	$T_{majorAxis}=||v_{major}||$ where $v_{major}$ is the eigenvector corresponding to the maximum eigenvalue	
Tumor Minor Axis Length $T_{minorAxis}$	The shortest linear dimension of the tumor along its primary axis.	$T_{minorAxis}=||v_{minor}||$ where $v_{minor}$ is the eigenvector corresponding to the minimum eigenvalue	
Tumor Flatness $T_{flat}$	Used to describe whether the shape of the 3D tumor region tends to be more flat.	$T_{flat}=T_{majorAxis}/T_{mediumAxis}$ where $T_{mediumAxis}$ is the medium linear dimension of the tumor along its primary axis.	
**Tumor-Vessel Spatial Location Features**			
Encasement angle* $\theta_{encase}$	On a blood vessel, calculate the maximum proportion of the vessel circumference that is in contact with the tumor in cross-sectional, coronal, and sagittal slices.	$\theta_{encase}=\underset{a\in \left\{x,y,z\right\}}{max}\left(\underset{s\in \left[0,d\right]}{max}\left(\frac{L_{s}^{a}}{C_{s}^{a}}\right)\right)$ where $C_{s}^{a}$ and $L_{s}^{a}$ denotes the vessel circumference and its part in contact with the tumor at the s-th cross-section perpendicular to axis a and the length in contact with the tumor	According to NCCN guidelines, an increase in the degree of tumor encasement around blood vessels is associated with an increased risk of vascular involvement.
Minimum Vessel Radius Distance Product* $D_{min}$	Minimum, maximum, mean and standard deviation of the sequence extracted along the vascular skeleton consisting of the product of the vascular radius and the shortest distance between vascular skeleton voxels and the tumor. The minimum value in this sequence.	$D_{min}=min\left(D^{n}\right)$ where $D^{n}$ is a list containing the product of vessel radius and tumor distance at every voxel from a skeleton edge with a length of n voxels	Both decrease in vessel radius and decrease in the distance between vessels and the tumor can lead to increase the risk of involvement. Taking the product of these two factors makes this characteristic easier to capture.
Maximum Vessel Radius Distance Product* $D_{max}$	...	$D_{max}=max\left(D^{n}\right)$	
Mean Vessel Radius Distance Product* $D_{mean}$	...	$D_{max}=\frac{\sum (D^{n})}{n}$	
Vessel Radius Distance Product Range* $D_{range}$	...	$D_{range}=D_{max}-D_{min}$	
Vessel Radius Distance Product Standard Deviation* $D_{std}$	...	$D_{std}=\sqrt{\frac{\sum ({\left(D-D_{mean}\right)}^{2})}{n}}$	
Vessel Radius Distance Product Ratio* $D_{ratio}$	Divide the vascular skeleton into two groups based on their distance from the tumor within the ROI and outside the ROI. Calculate the ratio of the mean product of radius and distance for the two groups	$D_{ratio}=\frac{\left(\sum {}_{p\in ROI_{out}}D^{P}\right)/n_{out}}{\left(\sum {}_{p\in ROI^{D^{p}}}\right)/n}$ where ROI and $ROI_{out}$ denotes the range of skeleton voxels inside and outside the ROI, n and $n_{out}$ denote the number of skeleton voxels inside/outside the ROI	Vessels closer to the tumor are more likely to be affected by tumor compression. Taking the ratio of distances between regions of interest (ROI) inside and outside can reflect the degree of narrowing deformation caused by the tumor within the ROI area.
Contact Area* $C_{area}$	Number of voxels adjacent to the tumor on the vessel surface.	$C_{area}\;=\;|\left\{v\;\in \;S\;\vee \;\exists t\;\in \;Tum,\;||\;\nu \;-\;t\;||\;\leq \;\sqrt{3}\right\}|$ where S denotes the surface of target vessel, and Tum denotes the tumor voxels	The involved cases might have more voxels contact with tumor.
Contact Length* $C_{length}$	Number of skeleton voxels with mappings to surface voxels.	$C_{length}=\left|\left\{k\in Skele\vee \exists v\in Surf,f\left(v\right)=k\right\}\right|$ where Surf and Skele respectively denote the surface and skeleton voxels of target vessel	According to clinical diagnostic experience, an increase in the length of contact between the tumor and blood vessels increases the risk of vascular involvement.

Features with “*” denotes the anatomically guided features (14 in total) extracted by designed method;

Features without “*” are tumor morphological features (27 in total) extracted by pyradiomic tool, and the most representative ones (8 in total) are presented.

Details about mentioned vascular skeleton see Appendix D.

In this study, 61 patients underwent radical resection, of which 40 patients (46.0%) underwent pancreaticoduodenectomy and 21 patients (24.1%) underwent distal pancreatectomy. 26 patients (29.9%) underwent palliative surgery/exploratory laparotomy. Partial vein resection and reconstruction were performed in 10 patients (16.4%) who underwent radical resection.

### Preoperative assessment of vascular involvement

The results of the consistency analysis of tumor-vessel relationship assessments by the two radiologists, evaluated using Cohen’s kappa, showed moderate agreement for SMA (0.50), strong agreement for CA (0.78), CHA (0.78), and SMV (0.66), and very strong agreement for PV (0.84).

### Regression models of vascular involvement prediction

As shown in (Fig. 4(C)), the defined 229 features were selected and merged to construct a regression model for vascular involvement prediction. First, $\theta_{encase}$ (calculated as shown in Fig. 4(B)), reflecting the tumor-vessel circumferential contact degree, was included in the model.

Next, we selected the 4 most strongly associated common features from 13 vascular morphological features and tumor-vessel spatial relationship features on the PV and SMV vessels . These features, which are most correlated with vascular stenosis, deformity, and occlusion, include $R_{std}$, $D_{max}$, $D_{std}$, $D_{ratio}$ (see Table 2), and were then regressed to form vascular deformation risk score ( $RS_{deform}$).

(1) $$RS_{deform}=\frac{1}{1+exp\left[-\left(\beta_{0}+\beta_{1}\cdot R_{std}+\beta_{2}\cdot D_{max}+\beta_{3}\cdot D_{std}+\beta_{4}\cdot D_{ratio}\right)\right]}$$

where, $\begin{array}{c} \beta_{0}=-1.098\;\;\;\beta_{1}=0.663 \end{array}$ $\begin{array}{c} \beta_{2}=-1.086\;\;\;\;\beta_{3}=-0.745\;\;\;\; \end{array}$ $\beta_{4}=2.395$

The algorithm^[1]^ for $RS_{deform}$ indicates that the likelihood of vascular involvement increases when (a) the vascular morphology deviates more from a cylindrical shape, or (b) there is localized narrowing of the vessel near the tumor (with a smaller radius compared to other segments).

$RS_{deform}$ can characterize the degree of vascular deformation by describing the changes in vascular radius and the vessel-tumor distance along the vascular trajectory, thereby predicting the risk of tumor involvement in the vessel.

Next, from the high-throughput feature set extracted using pyradiomics (27 tumor morphological features and 188 texture features), we selected the two features most strongly correlated with the reference standard, $T_{flat}$ and (see Table 2). These were then regressed to form tumor risk score ( $RS_{tumor}$).

(2) $$RS_{tumor}=\frac{1}{1+exp\left[-\left(\beta_{0}+\beta_{1}\cdot T_{flat}+\beta_{2}\cdot T_{sphere}\right)\right]}$$

where,

$\begin{array}{c} \beta_{0}=1.526\;\;\;\;\beta_{1}=7.093\;\;\;\;\beta_{2}=-9.826 \end{array}$

The algorithm for $RS_{tumor}$^[2]^ indicates that as the tumor morphology deviates more from a spherical shape (more irregular or flattened), the likelihood of vascular involvement increases.

The detailed feature selection is described in Supplemental Digital Content Appendix E, available at: http://links.lww.com/JS9/E676. Finally, regression models predicting vascular involvement were constructed for each vessel. The features $\theta_{encase}$, $RS_{tumor}$, and $RS_{deform}$ (veins only) were included in the model, with intraoperative vascular involvement serving as the reference standard.

**Figure 5.**
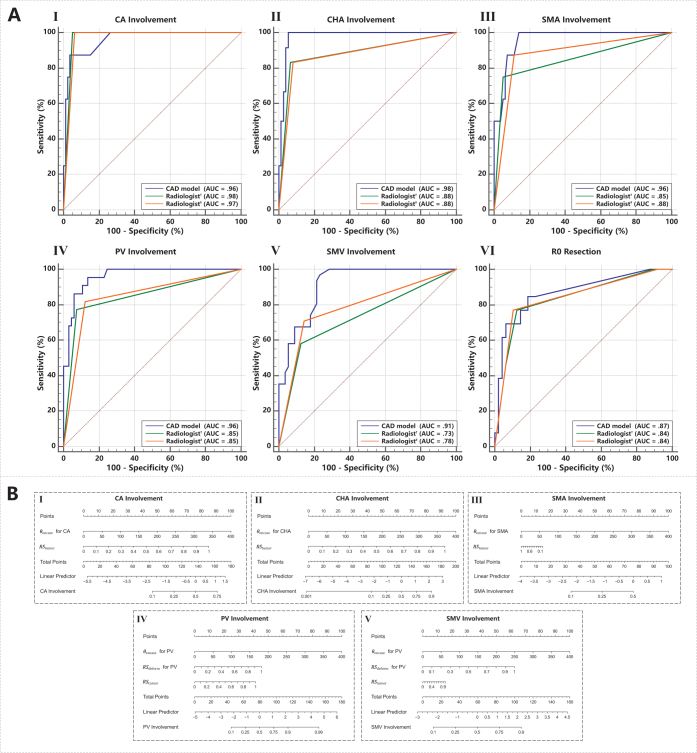
The results of regression models in proposed CAD model. **(A)** Comparison of ROC curve and AUC of assessments and predictions between CAD model and consensus of radiologists (^†^ and ^‡^ for the criterion of encasement and abutment/encasement respectively). **I, II, III, IV, V** represent the assessment of vascular involvement on five vessels (CA, CHA, SMA, PV, SMV); **VI** represents the prediction task of resection margins R0. **(B)** Nomograms of vascular involvement regression models for prediction of vascular involvement on each vessel. **I, II, III, IV, V** respectively represent the CAD model of CA, CHA, SMA, PV and SMV.

We report the classification performance of the model (Sensitivity, Specificity, PPV, NPV) and ROC analysis (AUC) in Table 3, and the calibration curves and DCA (Decision Curve Analysis) curves are presented in Supplemental Digital Content Appendix G, available at: http://links.lww.com/JS9/E676. The ROC curves are shown in (Fig.5 (A)). The results indicate that the CAD model’s AUC values for predicting vascular involvement in CHA, SMA, PV, and SMV are superior to those of the radiologists, with statistically significant differences for PV and SMV (P < 0.05). For predicting CA involvement, the model’s AUC is comparable to that of the radiologists. Specifically, when using “encasement” as the predictor, the CAD model’s sensitivity is comparable to that of radiologists (CA, CHA) or partially higher (SMA, PV, SMV), while its specificity is comparable (CA, CHA) or partially lower (SMA, PV, SMV). When using “encasement/abutment” as the predictor, the CAD model’s sensitivity and specificity are comparable to those of radiologists (P > .05).

**Table 3:** Radiologists and CAD Model Predictions for Vascular Involvement and R0 Resections

	CAD model	Radiologist^b^	*P*^a^	Radiologist^c^	*P*^a^
CA					
Sens.	.88(7/ 8)	.88(7/ 8)	.48	1.00(8/ 8)	>.99
Spec.	.96(76/79)	.99(78/79)	.48	.96(76/79)	>.99
PPV	.70(7/10)	.88(7/ 8)		.73(8/11)	
NPV	.99(76/77)	.99(78/79)		1.00(76/76)	
AUC	.96(.89,.99)	.98(.91,.99)	.62	.97(.90,.99)	.79
CHA					
Sens.	1.00(12/12)	.83(10/12)	.48	1.00(12/12)	>.99
Spec.	.93(70/75)	.96(72/75)	.62	.95(71/75)	>.99
PPV	.71(12/17)	.77(10/13)		.75(12/16)	
NPV	1.00(70/70)	.97(72/74)		1.00(71/71)	
AUC	.98(.92,.99)	.88(.79,.94)	.10	.88(.78,.93)	.08
SMA					
Sens.	1.00(8/ 8)	.50(4/ 8)	.13	1.00(8/ 8)	>.99
Spec.	.85(67/79)	.97(77/79)	.004^e^	.84(66/79)	>.99
PPV	.40(8/20)	.67(4/ 6)		.38(8/21)	
NPV	1.00(67/67)	.95(77/81)		1.00(66/66)	
AUC	.96(.89,.99)	.85(.75,.91)	.16	.88(.79,.94)	.21
PV					
Sens.	.95(21/22)	.73(16/22)	.07	.91(20/22)	>.99
Spec.	.85(55/65)	.95(62/65)	.02^d^	.85(55/65)	.68
PPV	.68(21/31)	.84(16/19)		.67(20/30)	
NPV	.98(55/56)	.91(62/68)		.96(55/57)	
AUC	.96(.89,.99)	.85(.75,.91)	.009^e^	.85(.75,.91)	.006^e^
SMV					
Sens.	1.00(31/31)	.71(22/31)	.007^e^	.94(29/31)	.48
Spec.	.75(42/56)	.89(50/56)	.013^d^	.77(43/56)	>.99
PPV	.69(31/45)	.79(22/28)		.69(29/42)	
NPV	1.00(42/42)	.85(50/59)		.96(43/45)	
AUC	.91(.83,.96)	.73(.62,.81)	<.001^e^	.78(.68,.86)	.003^e^
Resection					
Sens.	.85(11/13)	.77(10/13)	>.99	.77(10/13)	>.99
Spec.	.81(39/48)	.88(42/48)	.55	.90(43/48)	.34
PPV	.55(11/20)	.63(10/16)		.67(10/15)	
NPV	.95(39/41)	.93(42/45)		.93(43/46)	
AUC	.87(.76,.94)	.84(.72,.92)	.70	.84(.73,.92)	.57

Note.—CA, CHA, SMA, PV, SMV represent tasks of predictions for intraoperative vascular involvement predictive, while Resection represent predictive task of R0 resection. CAD and Ra. respectively represent proposed the regression predictions of CAD model and consensus between two radiologists. Data in parentheses are numbers of patients except for AUC. Data in parentheses are 95% CIs only for AUC.

^a^ The P-values for sensitivity and specificity (derived from the McNemar test) and for AUC (derived from the DeLong test) assess the comparison between CAD model predictions and radiologist assessments. Both tests assume no significant difference between the two statistical results. A P-value < 0.05 rejects the null hypothesis, indicating a significant difference between the compared predictions.

^b^ Consensus assessment between two radiologists using “encasement” as the predictor.

^c^ Consensus assessment between two radiologists using “encasement/abutment” as the predictor.

^d^ Significance at the level of *P*<0.05

^e^ Significance at the level of *P*<0.01

The goodness of fit for the regression model was tested using the Hosmer-Lemeshow test (see Supplemental Digital Content Appendix F, available at: http://links.lww.com/JS9/E676 Table E3 for details). The weights of features in the regression model are presented in Table 4 and are visualized as a nomogram in (Fig. 5(B)), which explicitly illustrates the impact proportions of individual imaging features in predicting the involvement risk for each specific vessel.

**Table 4:** Features Included in Vascular Involvement Regression Models

Vessel	Feature	p	Odd Ratios	95% CI
CA	$RS_{tumor}$	.50	9.22	0.02, 5.49E + 3
	$\theta_{encase}$	**.002^b^**	1.01	1.01, 1.02
CHA	$RS_{tumor}$	.15	1.01E + 4	0.09, 1.10E + 7
	$\theta_{encase}$	**.001^b^**	1.02	1.00, 1.02
SMA	$RS_{tumor}$	.25	0.45	0.01, 8.74
	$\theta_{encase}$	**.001^b^**	1.02	1.01, 1.03
PV	$RS_{tumor}$	.15	13.84	0.40, 4.96E + 2
	$\theta_{encase}$	**.008^b^**	1.02	1.00, 1.03
	$RS_{deform}$	**.03^a^**	17.80	1.36, 232.13
SMV	$RS_{tumor}$	.55	2.03	0.18, 23.99
	$\theta_{encase}$	**.009^b^**	1.01	1.00, 1.02
	$RS_{deform}$	**.03^a^**	**17.54**	**1.43, 2.15E + 2**

Note**.—CA, CHA, SMA, PV, SMV represent tasks of predictions for intraoperative vascular involvement predictive.**

^a^ **Significance at the level of P < 0.05**

^b^ **Significance at the level of P < 0.01**

The results indicate that $\theta_{encase}$ is an important risk factor for the involvement prediction of the five major vessels (P < .01). Additionally, $RS_{deform}$ is an important risk factor for the involvement prediction of veins (SMV, PV) (P < .05), whereas $RS_{tumor}$ is not for any vessel (P > .05). However, due to the high correlation between the features composing $RS_{tumor}$ and the reference standard of involvement, this score is retained in the regression model for predicting vascular involvement.

### Regression model of postoperative R0 resection prediction

We used the image features from Section 3.3 ( $RS_{tumor}$, $\theta_{encase}$, and venous $RS_{deform}$) as model inputs and the postoperative pathological margin results as the reference standard to construct a regression model for predicting postoperative R0 resection. The results indicate that the sensitivity, specificity, and AUC of the CAD model in predicting R0 resection show no significant statistical difference compared to the predictions by radiologists (Table 3).

**Figure 6.**
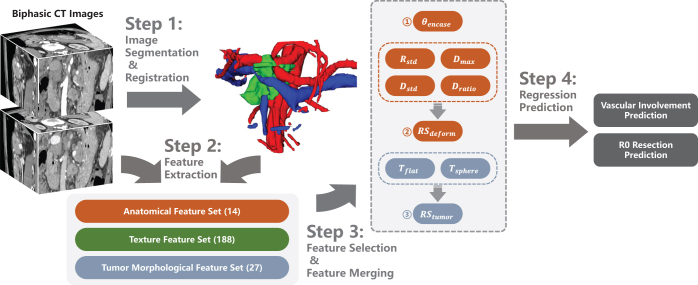
An example workflow of the proposed CAD model processing CT images of a PDAC patient. According to the consensus of radiologists, this patient is involved in CA, CHA, SMA, PV, and SMV, with no R0 resection achieved postoperatively. Using the best cutoff threshold of prediction probability threshold, the proposed CAD model accurately predicts all tasks except the involvement of CHA.

To illustrate the entire processing workflow of the proposed CAD model more clearly, (Fig. 6) presents an example of a patient with PDAC. This figure shows the process from inputting CT images into proposed CAD model to predicting vascular involvement and R0 resection.

## Discussion

In this study, we extracted image features on CT images of patients with PDAC based on segmentation masks generated by a segmentation network trained on public datasets. And by feature selection and merging, we then constructed a CAD model to predict vascular involvement and R0 resection. The results of this study indicate that the tumor-vascular encasement angle ( $\theta_{encase}$) is a significant risk factor for predicting involvement of the five major peripancreatic vessels, and the vascular deformation risk score ( $RS_{deform}$) is a significant risk factor for predicting involvement of veins (PV, SMV). The AUC of proposed CAD model reached (CA) and partially exceeded (CHA, SMA, PV, SMV) the experienced pancreatic specialist radiologists’ predictions of prediction of vascular involvement, part (PV, SMV) of which showed significant statistical difference.

Specifically, the CAD model’s sensitivity is comparable to or higher than that of the radiologists, though it shows slightly lower specificity, which indicates complementary strengths between these two evaluations. The higher sensitivity of the CAD model substantially mitigates the risk of underestimating PDAC vascular involvement in clinical practice, potentially aiding in avoiding erroneous surgical decisions in unresectable or borderline resectable cases, and thus preventing surgeries for patients ineligible for resection. Furthermore, the CAD model was comparable to experienced pancreatic specialist radiologists in predicting R0 resection. This is of great significant assistance to radiologists who are not specialized in pancreatic diseases and surgeons. These results underscore the potential of proposed CAD model as a valuable diagnostic assistance tool for predicting PDAC vascular involvement and R0 resection.

Compared to other segmentation-based methods, our CAD model reduces the annotation cost and enhances imaging robustness by training the segmentation network on partially-annotated, multi-phase, multi-center public datasets. Notably, during model design and training, we prioritized generalizability by leveraging multi-center data (over single-center datasets) and adopting the state-of-the-art DoDNet framework, which has been validated to achieve higher segmentation accuracy than methods like nnUNet and Swin-Transformer^[33,34]^. (Detailed methodology and dataset descriptions are provided in Supplemental Digital Content Appendix B, available at: http://links.lww.com/JS9/E676.) In terms of feature extraction, the proposed CAD model comprehensively extracts 229 image features encompassing morphology, tumor-vessel spatial relationships, and texture to characterize vascular involvement.

Compared to previous studies that evaluated involvement of fewer PDAC vessels (e.g., SMV-PV, SMV, SMA)^[6,15,25]^, the proposed CAD model provides a comprehensive assessment of vascular involvement risk across multiple vessels, better aligning with clinical needs. Compared with studies extracting features via pipeline tools like pyradiomics^[11,15,17–19]^, our research additionally incorporates 14 anatomically designed features, which characterize tumor-vessel morphology and spatial relationships—these features form a tumor-vessel spatial feature set based on segmentation masks, independent of specific pixel value distributions. Thus, with accurate segmentation, they are minimally affected by domain discrepancies in non-homologous data, offering higher interpretability and facilitating clinical translation. This study shares similarities with Bereska JI et al.’s research^[11]^ in focusing on features related to tumor-vessel circumferential contact, but further quantifies features associated with vascular narrowing and deformation, providing novel predictive factors for vascular involvement. Moreover, to eliminate subjectivity in manual image interpretation, we used intraoperative findings as the reference standard, enhancing the study’s credibility.

This study has the following limitations. First, we observed that the vascular deformation risk score is an important risk factor for predicting vein involvement but not for arteries. A possible reason is that arterial vessel walls are generally thicker and have abundant elastic fibers in the middle layer, making them less prone to deformation. Moreover, PDAC vascular involvement typically requires initial invasion of the vascular adventitia followed by deeper infiltration (i.e., the middle and inner layers of the vessel). Since CT imaging mainly highlights the vascular lumen, subtle changes in the arterial vessel wall may not be easily detected with the current model. Future research should focus on enhancing the evaluation of arterial vessel walls, such as by incorporating a dark-blood vessel wall assessment system^[27]^. Second, Although a few studies have shown that tumors with more heterogeneous texture features tend to be more aggressive and may be associated with an increased risk of PV-SMV involvement^[22]^, in this study, texture features did not show statistical significance for the predicting vascular involvement. We speculate that this might be related to the heterogeneity of the included cases. Additionally, unlike some studies that used tumor segmentation on a single axial slice (2D analysis), this study employed 3D segmentation for texture feature analysis. Studies have shown that most texture features exhibit significant differences when comparing 2D and 3D segmentation^[35]^, which could also be a reason for the inconsistent results. Nonetheless, the changes in radiomics/texture features before and after neoadjuvant therapy for PDAC have certain potential in predicting the treatment response, resectability, and prognosis of the tumor. In the future, we can provide data support for other diagnostic tasks by combining the tumor texture and tumor-vascular spatial features before and after treatment. Third, the data used in this study were from a single center and lack of external validation, which may limit the generalizability of the results. In the next phase of our study, we plan to 1) conduct multi-center cooperation to integrate highly heterogeneous data sets; 2) validation using an external dataset; 3) integrating multi-center data to optimize the robustness and generalization of the model by covering a wider spectrum of device parameters, operating procedures, and patient subgroups.

In summary, we developed a computer-aided diagnostic model that performs automatic segmentation of PDAC and adjacent vessels, and quantitatively extracts, selects, and analyzes features related to perivascular morphology, tumor morphology, and tumor-vascular spatial relationships. This model predicts vascular involvement and R0 resection in PDAC and can serve as an objective, reproducible, and precise diagnostic assistance tool.

## Data Availability

The datasets generated and/or analyzed during the current study are not publicly available due to the presence of sensitive patient information that could compromise research participant privacy. Data may be available from the corresponding author upon reasonable request, subject to ethical approval.

## Research registration unique identifying number (UIN)

We have successfully registered our study in the Chinese Clinical Trial Registry (ChiCTR), a publicly accessible platform approved by the World Health Organization (WHO). The registration details are as follows: Registry Used: Chinese Clinical Trial Registry (ChiCTR), Registration Number: ChiCTR2100042415. Hyperlink to Registration: https://www.chictr.org.cn/showproj.html?proj=120179&u_atoken=f1c81db64f86bb3569e4c1734f51b696.&u_asig=0a472f4317409905700913654e011a The registration ensures that our study adheres to international standards for transparency and accountability in clinical research. All relevant information about the study, including its objectives, methodology, and participant information, has been disclosed in the registry. If you require any additional information or clarification, please contact us.
